# Dental implants in patients suffering from systemic sclerosis: a retrospective analysis of clinical outcomes in a case series with 24 patients

**DOI:** 10.1186/s40729-021-00398-9

**Published:** 2021-12-27

**Authors:** Jochen Jackowski, Frank Peter Strietzel, Nicolas Hunzelmann, Parwana Parwani, Angelika Jackowski, Korbinian Benz

**Affiliations:** 1 Department of Oral Surgery and Dental Emergency Care, Faculty of Health, Witten/Herdecke University, Alfred-Herrhausen-Str. 45, 58448 Witten, Germany; 2 Charité Centre for Dentistry, Department Periodontology, Oral Medicine and Oral Surgery, Charité – University Berlin, Assmannshauser Str. 4-6, 14197 Berlin, Germany; 3 Department of Dermatology and Venerology, University of Cologne, Kerpener Str. 62, 50937 Cologne, Germany

**Keywords:** Systemic sclerosis, Rare disease, Immunosuppression, Dental implant, Implant survival rate

## Abstract

**Purpose:**

Patients with systemic sclerosis (SSc) often suffer from premature tooth loss. This is a retrospective case series of patients with systemic sclerosis who were treated with dental implants.

**Methods:**

SSc patients treated with at least one dental implant between 5 August 1998 and 31 December 2018 were included in this long-term retrospective study. The primary study variables were the plaque index (PLI), sulcus bleeding index (SBI), peri-implant pocket depth (PPD) and interincisal distance (ID). The test for marginal homogeneity analysed whether the SBI and PLI values changed between examination and follow-up. A linear regression was performed for the PPD measurement. The rank correlation coefficient compared the SBI with the PLI and the PPD with the PLI. The survival rate data for the implants were analysed by the Kaplan–Meier procedure. P < .05 was considered significant.

**Results:**

Twenty-four patients [(age: mean 59.6 years (SD ± 13.08)] received a total of 72 implants. ID resulted in a mean value of 29.54 mm (SD ± 6.4 mm). The mean value of the PPD was between 2.4 mm and 2.8 mm. A comparison of the SBI with the PLI and the PPD with the PLI showed a significantly positive correlation between the SBI and the PLI and between the PPD and the PLI. The correlation between the PPD and the PLI (Spearman rho: 0.36, *p* < 0.001) was less pronounced than that between the SBI and the PLI (Spearman rho: 0.61, *p* < 0.001). Kaplan–Meier analysis showed a post-10-year implant survival rate of 87.6% (95%-KI: 75.5–94.0).

**Conclusion:**

Implant-supported oral rehabilitation can be carried out and maintained successfully in SSc patients.

## Background

The term systemic sclerosis (SSc) is assigned to a complex of inflammatory and fibrotic diseases referred to as collagenoses. SSc is a chronic inflammatory disease that affects vascular connective tissue and accompanies localised or generalised dermal fibrosis. The pooled overall prevalence of SSc was 17.6 (95% CI 15.1, 20.5) per 100,000 inhabitants, with a pooled overall incidence of 1.4 (95% CI 1.1, 1.9) new manifestations per annum and 100,000 inhabitants. The gynaecotropism for SSc amounts to 5:1 [1].

The disease onset peak occurs between the ages of 30 and 50 or between the ages of 45 and 65, depending on the literature [2]. Limited systemic scleroderma (ISSc) can be differentiated from diffuse systemic scleroderma (dSSc) as two fundamental clinical subgroups. Differentiation is possible with regard to the progress of the disease, the involvement of the internal organs and the specific antibodies produced [3].

The diagnosis criteria, which were originally defined by the American College of Rheumatology (ACR), were subjected to further delineation some years ago in cooperation with the European League against Rheumatism (EULAR). These now comprise clinical, clinical chemistry and immunoserological data that are weighted on the basis of their value, thus forming a summation score [4]. A total weighting of > 9 is evaluated as representing a certain diagnosis of SSc. Pachydermia that affects the hands and extends proximal from the metacarpophalangeal joint is deemed to suffice for the classification of a patient as a scleroderma patient. The differential diagnostics include autoantibody diagnostics with anti-centromer, anti-topoisomerase I (Scl-70) and anti-RNA polymerase III antibodies, which are typical for SSc. When combined with findings from capillary microscopy, the disease can be determined not only earlier but also with greater sensitivity (Table 1) [5].

**Table 1:** Current classification of the SSc [66]

Items	Subitems	Weighting (Points)
Pachydermia of the hands, proximal metacarpophalangeal joint (adequate criterion)		9
Pachydermia of the fingers (only the highest points)	Swelling of the fingers (puffy fingers) Sclerodactyly	2 4
Lesions to the fingertips (only the highest points)	Ulcers of the fingertips Scars on the fingertips (pitting scars)	2 3
Teleangiectasias		2
Abnormal peryonchial capillaries		2
Pulmonary participation (max. 2 points)	Pulmonary arterial hypertension Interstitial lung disease	2 2
Raynaud´s phenomenon		3
SSc-typical autoantibodies (max. 3 points)	Anti-centromere Anti-Topoisomerase I (Scl70) Anti-RNA Polymerase III	3

Dermatosclerosis is a characteristic symptom of SSc, whereby an initial manifestation of dermatosclerosis affects the fingers [6]. Those afflicted report a rubor (redness) and swelling of their fingers, referred to as “swollen hands” or “puffy fingers”. These indications are interpreted as early symptoms, as in some cases, they can manifest themselves years in advance of the actual diagnosis.

**Fig. 1:**
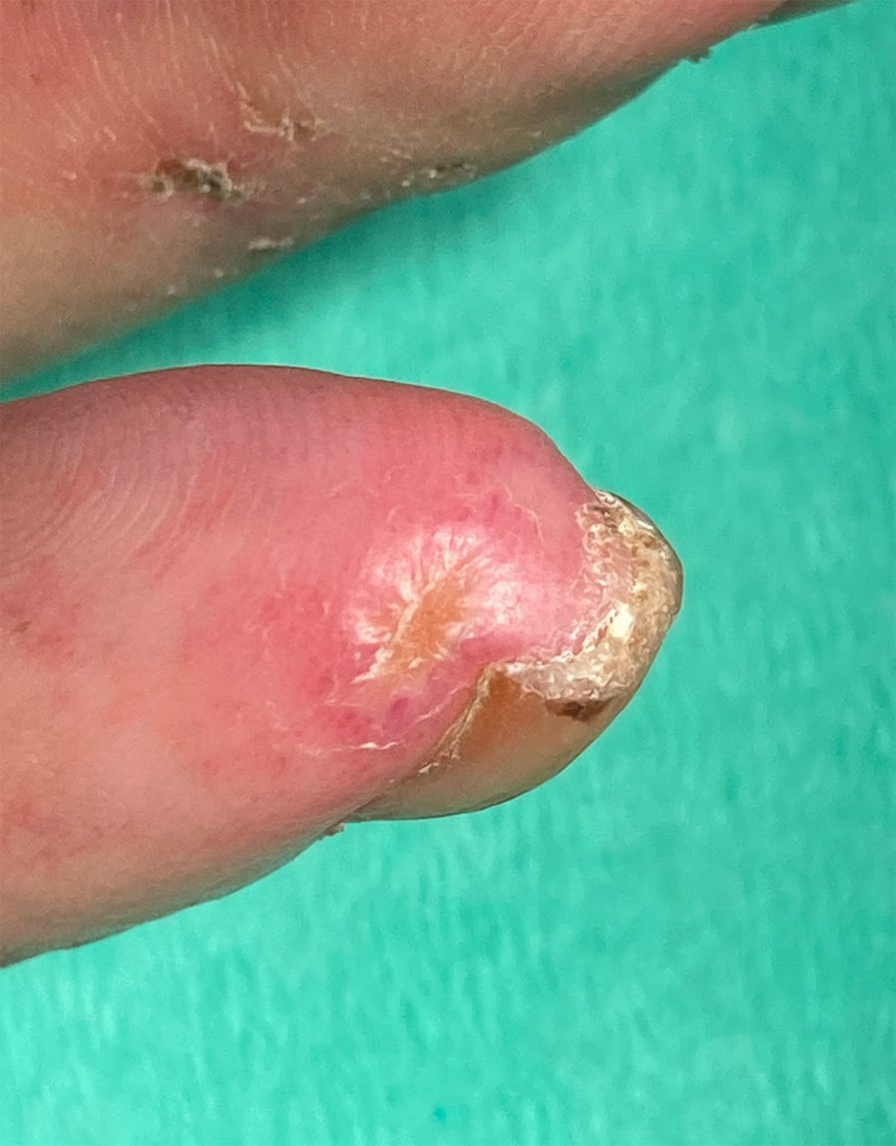
Ulceration of the fingertip in an 80-year-old woman with SSc

Secondary Raynaud´s disease occurs frequently as a result of circulatory disturbances and is found in almost 90% of all cases. It is often the initial symptom of SSc and is the result of disturbed vascular regulation with primarily vasoconstrictive influences (“Tricolour phenomena”: acral white, blue and red colouring) [6]. Another symptom of vascular involvement is digital ulceration, which occurs in up to 50% of patients and is characterised by rat-bite-like necrosis of the fingertips (Fig. 1). Contractures and sclerosis of the fingers result in so-called “Madonna fingers” and accompany a loss of the soft tissue mantle on the fingertips, all of which lead to restricted mobility.

The internal organs that are decisively affected are the heart, lungs and kidneys. The clinical symptoms depend on the speed and the extent to which the organ is affected. They have an effect on the quality of life and the survival prognosis of the patient [7–9].

In pathogenic terms, the origin and progression of SSc are caused by a triad of changes, namely, extensive changes resembling vasculopathy, humoural and cellular immunological anomalies and progressive and excessive fibrosis affecting the skin and the internal organs [10–21]. A clear temporal sequence or prioritisation is not possible. A genetic predisposition for the formation of SSc is the subject of discussion [22].

To date, no pathogenically oriented therapy for SSc exists that is able to delay or prevent the course of the disease or the internal organs from being affected. The focus until now has hence primarily been directed to the treatment of symptoms [6]. Relevant medicinal therapeutics can be subdivided into immunosuppressive/anti-inflammatory therapy, the treatment of vascular complications and antifibrotic therapy. Autologous stem-cell transplantation is also conducted at specialised centres [23, 24].

**Fig. 2:**
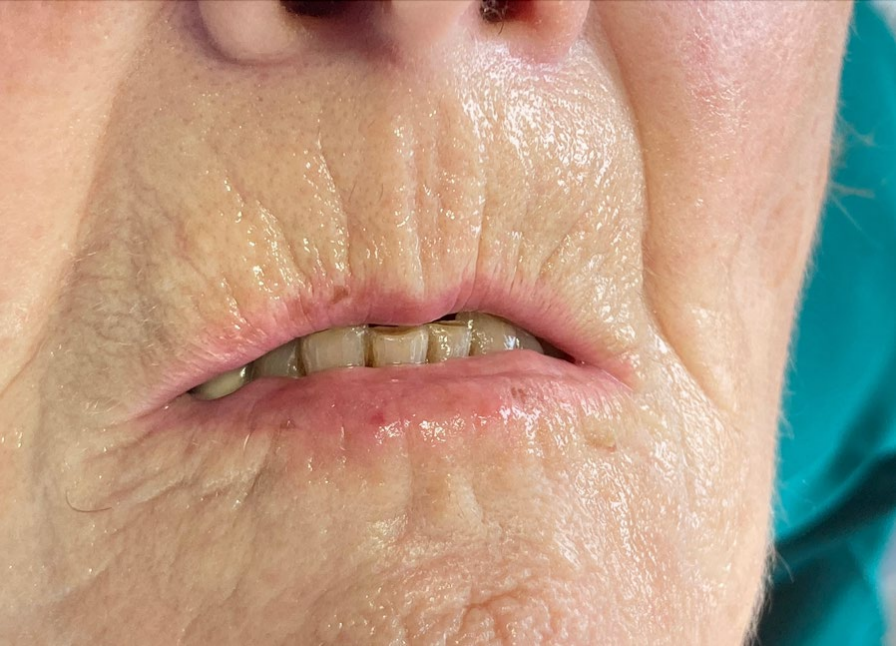
Typical radial folds (“tobacco pouch mouth”) in a 74-year-old woman with SSc

Not only Raynaud’s symptoms and the pathognomonic physiognomy (a pointed nose, taut skin, amimia) but also the detection of changes to the oral cavity play important roles in the early diagnosis of SSc. The shortening and thickening of the frenulum of the tongue, scleroglosson, is one of the earliest symptoms of SSc [25]. In the orofacial area, the lips are most frequently affected by changes [26]. The cutaneous, mucous and muscular parts can all be sclerosed [27]. If the M. orbicularis oris is included in this process, the results are the typical radial folds that surround the month, an appearance often referred to as “tobacco pouch mouth” (Fig. 2), and a considerable restriction to the opening of the mouth function, also referred to as microstomia [28, 29]. Multivariate regression analyses confirmed SSc as a significant independent predictor of developing a reduced incisal edge distance (IED) [30].

Patients with SSc have been shown to have a lower gingival bleeding index, a higher periodontal attachment loss and a shallower pouch depth than healthy controls [31]. This higher periodontal attachment loss among SSc patients has also been confirmed by an additional study, which provided indications of a dependency between SSc status and periodontitis [32]. In another study, SSc patients (*n* = 80) complained more frequently of dysgeusia, dysphagia and stomatodynia than did a control group without rheumatological anamnesis (*n* = 80) [33].

In 2015, 163 SSc patients and 231 controls participated in the Canadian Systemic Sclerosis Oral Health Study IV, the aim of which was to compare radiological changes related to scleroderma with radiological abnormalities/variations from the norm among the normal population [34]. A widened periodontal gap was discernible to a significantly greater extent in SSc patients than in the control group. The greatest differences between the two study groups were found between the premolar and molar areas. This symptom, referred to as the Stafne sign, is deemed to be one of the initial early symptoms of the disease and is also believed to indicate the pathway of the disease in pathognomic terms [35]. The osseous erosions that White et al. (1939) described were detected to a more frequent extent on the condyles, on the processus coronoideus and on other regions of the mandible and the maxilla of SSc patients than was the case in the control group [34, 36].

The objective of the present study was to conduct an in-dwelling analysis of implants in patients suffering from SSc that resulted in a reduced opening of the mouth and restricted mobility and tactility in the area of the fingers/hands. Data concerning the specific clinical findings of probing depth, biofilm accumulation and bleeding on probing in the peri-implant sulcus area were collected from such patients for expressive, generally accepted and practical predictions to be made with regard to future losses of implants in individuals suffering from SSc.

## Materials and methods

### Selection of patients and implants

By completing the anamnesis form and signing it, the patients declared their consent for the processing of the data for scientific purposes. The study was approved by the local Ethics Commission at Witten/Herdecke University (ethics vote No. 57/2017) and prepared in accordance with the STROBE guidelines [37]. The follow-ups included all of the patients with SSc who had received implants at the Department Oral Surgery and Dental Emergency Care at Witten/Herdecke University between 5 August 1998 and 31 December 2018. The patients were not specifically asked to come to the clinic for this purpose to avoid exposing them to any additional burdens. The data were collected as part of the regular maintenance care program.

All of the patients who participated in this retrospective clinical study had been diagnosed with SSc according to the ACR/EULAR diagnosis criteria [4].

In addition to diverse organ involvement and orofacial manifestations, the patients suffered from Raynaud´s disease, including “swollen hands” or “puffy fingers”, necroses of the fingertips with an appearance similar to rat bites, calcinosis cutis affecting the fingers and/or “Madonna fingers”.

The inclusion criteria for implant-supported care were as follows:

- restoration of the chewing function only possible with implants;
- adequate local bone supply without the use of advanced or simultaneous augmentation proceedings;
- screwability of the supraconstruction;
- high degree of motivation on the part of the patients;
- responsible physical burden by surgical intervention.

The integration time for all of the implants, on average, was four months in the maxilla and three months in the mandible. Where possible, regular follow-up examinations were carried out after completion of surgical and prosthetic therapy. The initial collection of the clinical parameters that formed the basis of this retrospective study took place at an interval of 14 days after the implant prosthetic restoration had been inserted (period 0 = baseline), whereas the last collection was after 120 months (period 0 to 120). In the first year, additional follow-up examinations were offered at intervals of three months (period 0 to 3), six months (period 0 to 6) and 12 months (period 0 to 12) so that the oral hygiene behaviour of the patients could be recorded at short intervals and support provided if necessary, for example, in the event of the existence of a disease-related reduced opening of the mouth and/or restricted hand and finger mobility. Both the implant surgery and the prosthetic treatment steps and the follow-up examinations were conducted by the same experienced oral surgeon. Functional loading was a condition for the survey of clinical parameters. They were collected within the scope of the clinical follow-up examinations.

The following standard parameters were collected within the scope of the clinical follow-up examinations:

1. Plaque Index (PLI): 0 = No detection of plaque, 1 = plaque only recognised by running a probe across the smooth marginal surface of the implant, 2 = plaque seen by the naked eye, 3 = plaque seen by the naked eye. Abundance of soft matter [38].
2. Sulcus Bleeding Index (SBI) (0 = no bleeding, 1 = isolated bleeding on probes, 2 = narrow paragingival line of blood, 3 = profuse bleeding).
3. Peri-implant pocket depth in mm (pocket probing depth: PPD) with a calibrated periodontal probe (Hu-Friedy, Chicago, United States) taken at four sides (mesial, buccal, distal, oral). To ensure the accuracy and reproducibility of each probing, the removal of the superstructures was obligatory [39].
4. Interincisal distance (ID) with a conventional calliper gauge (Kemmler, Mössingen, Germany). The vertical distance between the incisal edges of the incisors in the maxilla and the mandible is normally wider than 50 mm [29].

To obtain comparative periods between the first examination (period 0 = baseline) and the follow-up examinations, the implant-prosthetic follow-up was subdivided into periods of 3, 6, 12, 18, 24, 36, 48, 60, 72, 84, 96, 108 and 120 months, and the corresponding quantitative determination of the PLI, SBI, and PPD was allocated to these periods. Only the final examination was taken into account in the statistical analysis if a patient had received more than one follow-up examination of an implant position within the same period.

The probing depth in the implants was only measured after the prosthetic restorations had been removed. During evaluation of the measured PPD values, the examination period was subdivided into years (baseline, ≤ 1 year, ≤ 2 years, ≤ 3 years,… ≤ 10 years). As only one patient was still being subjected to probing depth measurements after 10 years, this study only encompassed those measurements that took place up to a period of 10 years after the baseline examination. If numerous probing depth measurements of an implant position had occurred within an annual period, only the result of the final examination was taken into account in the statistical analysis (analogous to the analysis of the PLI and the SBI). The probing depths were calculated on the basis of the mean value, the standard deviation, the median, and the minimum and maximum. The corresponding change (difference) for diverse periods starting at the baseline was also estimated in the form of a linear regression with a random effect.

The PLI and SBI were analysed over the course of time and compared with the baseline examination.

As with the probing depth measurement, the examination period was divided into 10 years, and the last measurement from each of the periods was taken into account. The maximum values from the four measuring points were included in the analysis for each of the patients, each of the implant positions and each of the examinations.

In another analysis, the SBI, PLI, PPD, and PLI were each compared with each other. A global test was also carried out to determine whether the three PLI groups differed with regard to the mean PPD. They were compared with each other in pairs.

The calculation of the survival probability for the inserted implant body was carried out during this case series.

Peri-implant follow-up radiological imaging was not carried out at annual follow-ups because of the disease-related changing general condition of the patients and their microstomia. An annual report of the mean radiologically measurable bone resorption was therefore not included in this study.

### Statistical analysis

The test for marginal homogeneity tested whether the SBI and PLI values changed between the two time points of the baseline examination and follow-up controls [0 + *n* (*n* = 3, 6..,120 months)].

The mean difference in probing depth within 1, 2… 10 years from baseline was estimated by random effects linear regression adjusted for implant position. The same method was used to test whether the mean probing depth at these timepoints was different from the baseline mean (Wald test).

The PLI and SBI determinations were made with a so-called 'ordered probit regression' to make a comparison with the baseline. The analysis included an adjustment for the position. If the estimated coefficient was significantly different from 0, then the baseline and the respective PLI or SBI value were considered to differ significantly with regard to the time point of the measurement. In the event of the coefficients being positive (negative), the probability of an increased PLI or SBI value was expected to be larger (smaller) than the baseline.

A linear regression (random effects linear regression) was also determined for the PPD measurement as a form of comparison with the baseline examination.

The rank correlation coefficient according to Spearman compared the SBI with the PLI and the STM with the PLI.

The survival rate data for the implants were analysed on the basis of the Kaplan–Meier procedure. The implant success rate was reported according to the criteria for implant success provided by Buser et al. [40].

A significance level of 0.05 was selected for the *p* value.

The evaluation was carried out using the statistics software Stata/IC 16.1 (StataCorp LLC, College Station, Texas, USA).

## Results

Twenty-four patients (age: mean 59.6 years (SD ± 13.08) received a total of 72 implants (66 implants in 22 women and six implants in two men). The determination of the interincisal distance resulted in a mean value of 29.54 mm (SD ± 6.4 mm). With regard to the supplier, 56 of the 72 implants were from Straumann (Basel, Switzerland) (52 × Regular Neck Tissue Level, 4 × Bone Level), 15 implants were from Thommen Medical SPI (Grenchen, Switzerland) and 1 implant was from Friadent (Frialit 2) (York, USA). The indication spectrum ranged from a single gap between the teeth to a complete lack of teeth. An inspection could not be carried out on one male patient and on three female patients with a total of seven implants, or only one follow-up examination was possible because two of the female patients died and the male patient and the third female patient were no longer able to participate in the recall for personal or health-related reasons. The remaining 20 patients comprised 19 females and one male with a total of 65 implants. Only 61 of these 65 implants could be included in the statistical analysis of the PLI, SBI and PPD, as no data were collected from 4 implants because of early loss. The mean observation period was 5.9 years, with a minimum of 0.7 and a maximum of 17.8 years. The IED, the SSc-related changes to the fingers and the hands and the respective prosthetic restoration are presented in Table 2.

**Table 2:** SSc patients with their ages at the time of surgery, sex, interincisal distance, SSc-related changes to the hands and fingers and prosthetic restoration (m = male; f = female)

Patient No	Age at the time of surgery	Gender	Medication* for the treatment of systemic sclerosis	ID (mm)	Fingers, hands	Prosthetic restoration
1	69	f	Corticosteroid (f)	42	Puffy fingers, calcinosis cutis in the area of the end phalanges, fingertip necroses	Bar restoration, removable prosthesis of the mandible
2	45	f	Corticosteroid (f)	38	Puffy fingers, fingertip necroses	Bar restoration, removable prosthesis of the mandible
3	65	f	Corticosteroid (f); MTX (p)	20	Puffy fingers, calcinosis cutis in the area of the end phalanges, fingertip necroses	Single crown
4	60	f	Analgesics/Anti-inflammatory drugs (p)	24	Puffy fingers, fingertip necroses	Screw-fixed dental bridge in the anterior maxilla
5	60	m	Corticosteroid (f)	30	Puffy fingers	Bar restoration, removable prosthesis of the maxilla
6	31	f	Corticosteroid (f); Analgesics/Anti-inflammatory drugs (f/p)	20	Madonna fingers calcinosis cutis in the area of the end phalanges, fingertip necroses	Bar restoration, removable prosthesis of the mandible, screw-fixed dental bridge in the anterior and posterior maxilla
7	65	f	Corticosteroid (f); MTX (p)	20	Sclerodactyly, fingertip necroses	Bar restoration, removable prosthesis of the maxilla Bar restoration, removable prosthesis of the mandible
8	66	f	Corticosteroid (f); MTX (p)	30	Puffy fingers, calcinosis cutis in the area of the end phalanges, fingertip necroses	Single crown
9	45	f	Corticosteroid (f); MTX (p)	24	Sclerodactyly, calcinosis cutis	Telescopic crowns with a removable prosthesis of the maxilla
10	55	f	Corticosteroid (p)	30	Puffy fingers, calcinosis cutis in the area of the end phalanges, fingertip necroses	Locators, removable prosthesis of the mandible
11	74	f	Analgesics/Anti-inflammatory drugs (p)	25	Sclerodactyly, calcinosis cutis in the area of the end phalanges, amputations in the area of the end phalanges	Bar restoration, removable prosthesis of the mandible
12	67	f	Corticosteroid (f)	25	Puffy fingers, calcinosis cutis in the area of the end phalanges	Bar restoration, removable prosthesis of the mandible
13	76	f	Corticosteroid (p); MTX (f)	29	Puffy fingers, calcinosis cutis	Bar restoration, removable prosthesis of the mandible
14	69	f	Analgesics/Anti-inflammatory drugs (p); Corticosteroid (p)	38	Puffy fingers, calcinosis cutis in the areas of the end phalanges, fingertip necroses	Bar restoration removable prosthesis of the mandible
15	38	f	Corticosteroid (f); MTX (p)	30	Puffy Fingers	Bar restoration, removable prosthesis of the maxilla, screw-fixed dental bridge in the anterior mandible
16	77	f	Corticosteroid (p)	34	Calcinosis cutis in the areas of the end phalanges, fingertip necroses, amputation in the area of the end phalanges	Bar restoration, removable prosthesis of the mandible
17	69	f	MTX (f)	25	Puffy fingers, calcinosis cutis	Bar restoration, removable prosthesis of the maxilla
18	77	m	Immunosuppressive (f)	39	Puffy fingers	Bar restoration, removable prosthesis of the mandible
19	30	f	Corticosteroid (f)	33	Puffy fingers, calcinosis cutis in the area of the end phalanges, fingertip necroses	Single crown
20	51	f	Analgesics/Anti-inflammatory drugs (p); MTX (p)	25	Madonna fingers, calcinosis cutis in the area of the end phalanges, fingertip necroses	Bar restoration, removable prosthesis of the maxilla
21	52	f	Corticosteroid (f); Immunosuppressive (p)	40	Calcinosis cutis in the area of the end phalanges, fingertip necroses	Single crown planned
22	58	f	Corticosteroid (f)	30	Madonna fingers, calcinosis cutis in the area of the end phalanges, fingertip necroses	Bar restoration removable prosthesis of the mandible
23	72	f	Corticosteroid (f)	30	Puffy fingers, calcinosis cutis in the area of the end phalanges, fingertip necroses	Bar restoration, removable prosthesis of the mandible
24	59	f	Corticosteroid (p); Immunosuppressive (p)	28	Madonna fingers, calcinosis cutis in the area of the end phalanges, fingertip necroses	Bar restoration, removable prosthesis of the mandible

*Depending on the stage of the disease (f = former/p = present: at the time of surgical and prosthodontic treatment and/or the observation period); *MTX * Methotrexate

**Fig. 3:**
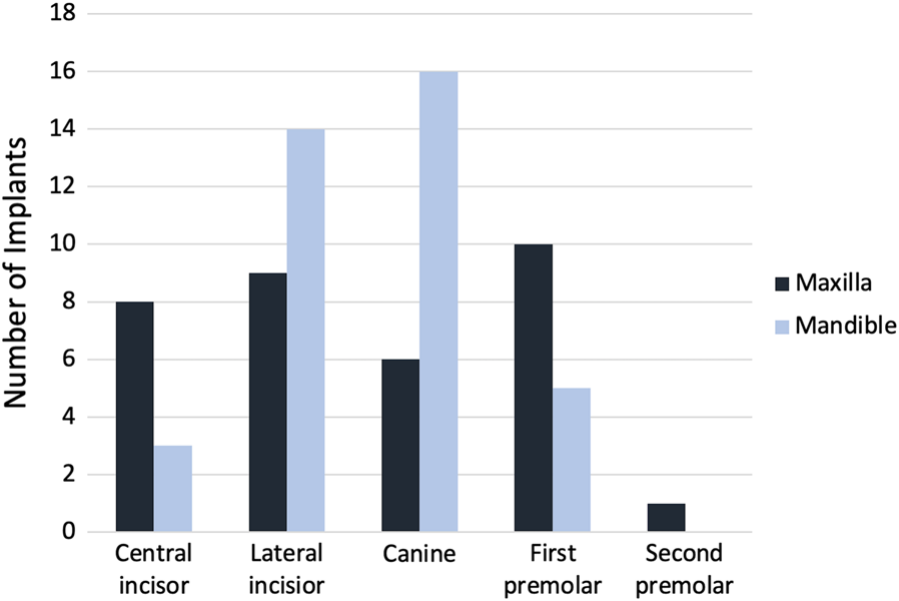
Distribution of the implant positions (*N* = 72) in 24 SSc patients

The distribution of the implant positions in the 24 patients is described in Fig. 3. All of the implants were inserted in the incisal and premolar areas of the maxilla and the mandibula.

The quantity of the implant types that were used, including the respective diameters and lengths, is shown in Table 3.

**Table 3:** Frequencies of the analysed implant types

Implant manufacturer/Type Implant diameter and length (mm/mm)	*N* (total = 65)	% Share	% Total
Straumann RN			70.7
3.3 × 10	12	18.5	
3.3 × 12	6	9.2	
4.1 × 8	2	3.1	
4.1 × 10	16	24.6	
4.1 × 12	9	13.8	
4.8 × 10	1	1.5	
Straumann BL			6.1
3.3 × 8	2	3.1	
3.3 × 10	1	1.5	
4.1 × 8	1	1.5	
Thommen Medical SPI			23.2
3.5 × 8	2	3.1	
3.5 × 9,5	1	1.5	
3.5 × 11	4	6.2	
4.2 × 9,5	4	6.2	
4.2 × 11	4	6.2	

*Four implants were not included in the statistical analysis because they were lost at an early stage

A total of 2592 PLI and SBI findings were collected, namely, values 0, 1, and 2 for the quantitative determination of the perimucosal plaque quantities and values 0, 1 and 2 for the Sulcus Bleeding Index. At the time that the examinations were carried out, plaque was not seen by the naked eye (Scale Value 3). Moreover, no narrow perimucosal lines of blood (SBI = 2) or profuse bleeding (SBI = 3) were discernible during the follow-up examinations. Only the last follow-up examination was taken into account with regard to patients who had received more than one examination of the same implant position during a follow-up period. In total, 1424 findings were recorded for the PLI and the SBI.

The examination period for the analyses that were conducted for the PLI (Table 4), the SBI (Table 5) and the PDM (Table 6) and the comparison with the initial examination (period 0 = baseline) were subdivided into years. As only one patient was still being subjected to probing depth measurements after 10 years, this study only included those measurements that took place up to a period of 10 years after the first examination (period 0 = baseline). The last measurement per implant and measuring point were taken into account within these periods. As discernible in Tables 4, 5 and 6, the number of implants or measuring points available at a follow-up examination fluctuated during the observation period of 10 years.

**Table 4:** PLI, 10 years – Description and comparison with the 1st examination (period 0 = Baseline). Number of implants (n)

					Comparison with Baseline*		
Period	*n*	0	1	2	Coeff	95%-KI	*p* value
Baseline	61	11 (18.0%)	34 (55.7%)	16 (26.2%)	–	–	–
≤1 Y	61	9 (14.8%)	24 (39.3%)	28 (45.9%)	0.77	0.27–1.27	0.002
≤2 Y	36	11 (30.6%)	10 (27.8%)	15 (41.7%)	0.16	− 0.42–0.74	0.582
≤3 Y	39	4 (10.3%)	17 (43.6%)	18 (46.2%)	0.69	0.10–1.28	0.022
≤4 Y	31	7 (22.6%)	16 (51.6%)	8 (25.8%)	0.05	− 0.53–0.63	0.858
≤5 Y	34	6 (17.6%)	15 (44.1%)	13 (38.2%)	0.60	− 0.00–1.21	0.051
≤6 Y	23	6 (26.1%)	9 (39.1%)	8 (34.8%)	0.55	− 0.13–1.23	0.111
≤7 Y	22	3 (13.6%)	14 (63.6%)	5 (22.7%)	0.78	0.07–1.50	0.031
≤8 Y	20	4 (20.0%)	9 (45.0%)	7 (35.0%)	0.68	− 0.07–1.44	0.075
≤9 Y	17	2 (11.8%)	10 (58.8%)	5 (29.4%)	0.78	− 0.02–1.58	0.057
≤10 Y	17	4 (23.5%)	10 (58.8%)	3 (17.6%)	0.48	− 0.31–1.26	0.232

^*^Random effects ordered probit

**Table 5:** SBI, 10 years—Description and comparison with the 1st examination (period 0 = Baseline). Number of implants (*n*)

					Comparison with Baseline*		
Period	*n*	0	1	2	Coeff	95%-KI	*p* value
Baseline	61	33 (54.1%)	24 (39.3%)	4 (6.6%)	–	–	–
≤1 Y	61	27 (44.3%)	30 (49.2%)	4 (6.6%)	0.43	− 0.12–0.99	0.125
≤2 Y	36	11 (30.6%)	20 (55.6%)	5 (13.9%)	1.16	0.49–1.84	0.001
≤3 Y	39	10 (25.6%)	24 (61.5%)	5 (12.8%)	1.21	0.55–1.87	< 0.001
≤4 Y	31	12 (38.7%)	19 (61.3%)	0 (0%)	0.82	0.13–1.51	0.020
≤5 Y	34	10 (29.4%)	23 (67.6%)	1 (2.9%)	1.08	0.41–1.75	0.002
≤6 Y	23	10 (43.5%)	13 (56.5%)	0 (0%)	0.67	− 0.10–1.45	0.086
≤7 Y	22	15 (68.2%)	7 (31.8%)	0 (0%)	− 0.01	− 0.84–0.82	0.977
≤8 Y	20	4 (20.0%)	16 (80.0%)	0 (0%)	1.63	0.80–2.47	< 0.001
≤9 Y	17	7 (41.2%)	10 (58.8%)	0 (0%)	0.89	0.05–1.73	0.039
≤10 Y	17	7 (41.2%)	10 (58.8%)	0 (0%)	1.02	0.17–1.87	0.018

*Random effects ordered probit

**Table 6:** PPD over time; comparison with the 1st measurement (0 = Baseline), the measurements being made within a period of 10 years. Number of measurement points (n)

						Comparison with Baseline*		
Period	*n*	Mean	Sd	Median	Min–Max	Difference	95% CI	*p* value
Baseline	200	2.6	0.6	3.0	0.0–4.0	–		
≤ 1 Y	146	2.5	0.6	2.5	0.0–4.0	− 0.02	− 0.11–0.07	0.654
≤2 Y	112	2.6	0.6	3.0	0.0–4.0	0.05	− 0.05–0.15	0.343
≤3 Y	124	2.5	0.6	2.0	0.0–4.0	0.04	− 0.05–0.14	0.401
≤4 Y	70	2.7	0.7	3.0	2.0–5.0	0.04	− 0.07–0.16	0.454
≤5 Y	118	2.5	0.7	2.0	0.0–4.0	− 0.06	− 0.16–0.04	0.224
≤6 Y	38	2.8	0.7	3.0	2.0–4.0	0.04	− 0.11–0.19	0.628
≤7 Y	70	2.4	0.6	2.0	2.0–4.0	− 0.06	− 0.18–0.05	0.277
≤8 Y	42	2.5	0.6	2.0	1.0–4.0	− 0.09	− 0.24–0.05	0.209
≤9 Y	52	2.6	0.6	2.0	2.0–4.0	0.12	− 0.01–0.25	0.074
≤10 Y	50	2.4	0.6	2.0	2.0–4.0	− 0.03	− 0.16–0.11	0.688

Last measurement per implant and measurement point within the period are taken into account

*Random effects linear regression adjusted for implant position

Table 4 shows the frequency of PLI values 0, 1 and 2 during the initial examination (baseline, 0) and during the various periods. A PLI value of 3 was not found during any of the examinations.

The PLI values for three of the ten recorded periods (1st year, 3rd year and 7th year) were significantly higher (Table 4) than those during the initial examination (period 0 = baseline).

Table 5 shows the frequency of SBIs 0, 1 and 2 during the initial examination (baseline, 0) and during the various periods. An SBI value of 3 did not occur in any of the examinations.

The analysis of the SBI values (Table 5) resulted in a determination of significantly increased SBI values in six of the ten recorded periods (2^nd^ year, 3^rd^ year, 4^th^ year, 5^th^ year, 8^th^ year and 9^th^ year) when compared with the initial examination (Period 0 = Baseline).

A total of 1022 probing depth measurements could be taken into account (Table 6). The maximum of each of the four measuring points (PPD) was included in the statistical analysis per patient, implant, and examination. The number of measuring points was reduced from 200 to 50 over a timeline of 10 years with annual measurements being made.

The mean value of the probing depths was between 2.4 mm and 2.8 mm.

An analysis of the PPD changes over a period of 10 years commencing with the baseline did not show any significant differences (Table 6).

A comparison of the SBI with the PLI (Table 7) and the PPD with the PLI (Table 8) indicate that a significantly positive correlation exists between the SBI and the PLI and between the PPD and the PLI in the patients with SSc recorded in this retrospective study. The correlation between the PPD and the PLI (Table 8) (Spearman rho: 0.36, *p* < 0.001) is, however, less pronounced than that between the SBI and the PLI [Spearman rho: 0.61, *p* < 0.001 (Table 7)]. This result occurs because the mean values of both of the groups PLI 0 (Mv 2.4) and PLI 1 (Mv 2.5) are almost identical (Table 9).

**Table 7:** SBI—PLI (all measurements)

SBI	PLI 0	PLI 1	PLI 2
	0 (*n* = 1104)	1 (*n* = 779)	2 (*n* = 709)
0	885 (80.2%)	405 (52.0%)	43 (6.1%)
1	219 (19.8%)	369 (47.4%)	580 (81.8%)
2	0 (0%)	5 (0.6%)	86 (12.1%)

Rank correlation coefficient according to Spearman rho: 0.61, *p* < 0.001

**Table 8:** PPD—PLI (all measurements)

PPD	PLI 0	PLI 1	PLI 2
	0 (*n* = 485)	1 (*n* = 390)	2 (*n* = 399)
0	2 (0.4%)	2 (0.5%)	1 (0.3%)
1	5 (1.0%)	2 (0.5%)	3 (0.8%)
2	308 (63.5%)	209 (53.6%)	74 (18.5%)
3	151 (31.1%)	158 (40.5%)	281 (70.4%)
4	18 (3.7%)	19 (4.9%)	40 (10.0%)
5	1 (0.2%)	0 (0%)	0 (0%)

**Table 9:** Description of the PPD on the basis of statistical key figures, depending on the PLI (all measurements)

PLI	Anzahl	Mw	Sd	Median	Min–Max
0	485	2.4	0.6	2.0	0.0–5.0
1	390	2.5	0.6	2.0	0.0–4.0
2	399	2.9	0.6	3.0	0.0–4.0

Rank correlation coefficient according to Spearman rho: 0.36, *p* < 0.001

In an analysis of the extent to which the three PLI groups (0, 1 and 2) differentiate with regard to the mean probing depth, no significant difference was found between PLI 0 and PLI 1 (Table 10).

**Table 10:** Difference between PLI 0, PLI 1 and PLI 2 with regard to the mean probing depth

Linear regression with a random effect:
globaler Test *p* < 0.001
PLI 0 vs. PLI 1: *p* = 0.933
PLI 0 vs. PLI 2: *p* < 0.001
PLI 1 vs. PLI 2: *p* < 0.001

Follow-up examinations were conducted on a total of 65 implants in 20 patients with regard to the survival rates of the implants. Sixty-one implants were included in the analysis, and no examinations were carried out on four of the implants because they were lost at an early stage.

Table 11 shows the duration of the functional period of the implant (the period between surgery and the last examination or between surgery and the loss of the implant), depending on its position.

**Table 11:** Function time (FU) of the implants in years (surgery to the last examination or until loss of the implant)

Position	n	Mw	Sd	Median	Min–Max
11	3	7.7	5.8	7.3	2.2–13.7
12	4	3.5	3.3	2.2	1.1–8.3
13	2	4.8	3.6	4.8	2.2–7.3
14	5	8.4	7.5	8.3	0.6–17.8
15	1	13.7		13.7	13.7–13.7
21	5	8.5	7.1	7.3	1.5–17.8
22	3	4.1	3.7	2.9	1.1–8.3
23	3	3.7	3.2	2.2	1.5–7.3
24	3	8.9	8.6	8.3	0.7–17.8
31	2	7.4	4.7	7.4	4.1–10.8
32	7	3.7	4.3	0.7	0.2–9.9
33	7	6.4	3.1	5.7	2.4–10.8
34	2	5.5	4.4	5.5	2.4–8.6
41	1	4.1		4.1	4.1–4.1
42	8	5.9	4.8	6.9	0.7–10.8
43	8	7.0	3.2	7.0	2.4–10.8
44	1	2.4		2.4	2.4–2.4
Overall	65	6.2	4.8	4.9	0.2–17.8
Period to the next examination	58	6.8	4.7	7.3	0.7–17.8
Time to loss	7	1.4	1.1	1.1	0.2–3.0

The period between surgery and the last examination of the implants that were not lost (*n* = 58) and the time between surgery and the loss of the implant (*n* = 7) are also shown.

The cumulative function time (FU-time) conforming to the sum of the times for the 65 implants was 401.7 years. When the seven losses were taken into account, the loss rate per annum (95%-KI) amounted to 1.74 (0.83–3.66%) (Table 12).

**Table 12:** Implant loss rates per year in percent

Implants	FU-Time (Years)	Losses	Loss Rate per Year in % (95%-KI)
65	401.7	7	1.74 (0.83–3.66)

The calculation of the survival rate after 1, 2 and 3 years indicated that the last implant loss took place after a period of 3 years. The estimated rate therefore remained constant at a level of 87.6% after 3 years (Table 13).

**Table 13:** Estimated survival rates after 1, 2 and 3 years (Kaplan–Meier estimations)

After… Years	Survival Rate in %	
1	Rate	95%-KI
1	95.4	86.4–98.5
2	92.0	81.8–96.6
3	87.6	75.5–94.0

The maximum examination period was 11 years for a female patient. The Kaplan–Meier plot shows the estimated survival rates (proportion of the implants without loss), depending on the period after surgery, and the timeline was restricted to 15 years.

**Fig. 4:**
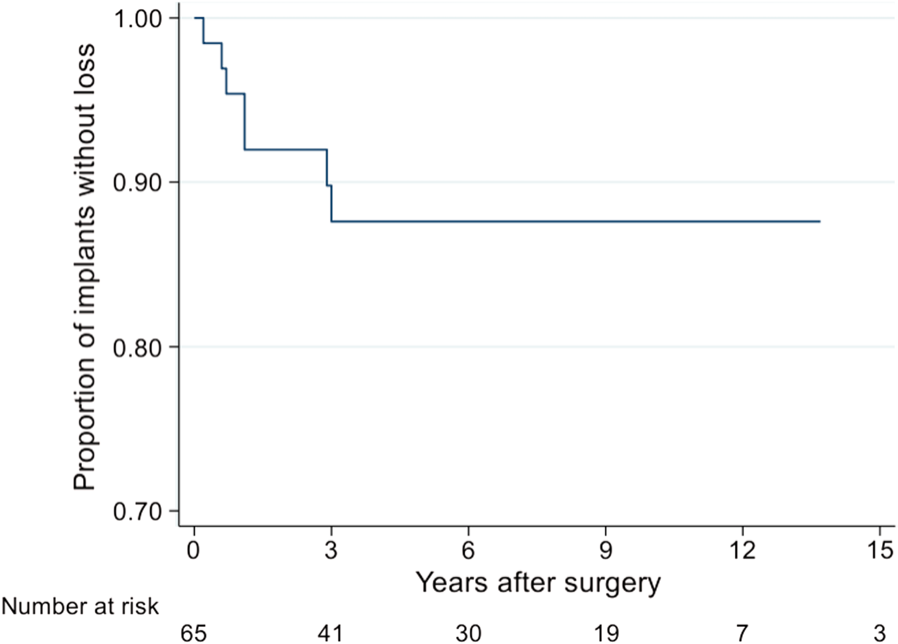
Estimated survival rate—Kaplan–Meier plot (period up to 15 years)

The numbers adjacent to 'Number at risk' indicate the number of implants having a correspondingly long survival rate. Of the initial 65 implants, 41 implants had a survival rate of at least 3 years, whereas 30 had a minimum survival rate of at least 6 years (Fig. 4).

Overall, the implant success rate was 89.2% (79.1–95.6; 95% CI).

## Discussion

Current knowledge regarding the therapeutic suitability of dental implants when treating patients suffering from rare diseases such as systemic sclerosis is exclusively based on clinical observations that have previously been made in single case reports and in small case series. The in-dwelling and survival duration of an implant are success criteria in implantology. High survival and success rates have been shown in connection with implant-supported oral rehabilitation over an observation period of more than 10 years [41]. Incalculable effects on the organism as a whole, potential risks for the osseointegration process, and a lack of statistically supported data have been the reasons for the cautious use of implants in patients suffering from rare diseases in the past. No generally valid implantological therapy guidelines exist for these patients at present, as evidence-based statements are not possible in view of the small number of cases that exist within each rare disease. In the event of there being coparticipation of the stomatognathic system, e.g., in the form of microstomia, xerostomia or inflammatory, ulcerous, erosive or bullous alterations to the oral mucosa or undesirable reactions to pharmaceutical drugs prescribed for the indicated anti-inflammatory or immunomodulation therapy, implants are the only therapeutic possibility for restoring chewing function, as far as some of the affected patients are concerned [42–44].

The approach of our study was a retrospective data evaluation, which we started with the successful implant-supported rehabilitation of a patient in 1998. At that time, it was not foreseeable that at least 24 SSc patients would be treated with implants in our institution over a period of two decades. This is the reason why we did not include a comparison group with healthy patients. A prospective approach was unrealistic, taking into account the rarity of the disease [prevalence of SSc 17.6 (95% CI 15.1, 20.5) per 100.000)], and for this reason, it has not been pursued further.

The literature includes reports concerning the successful rehabilitation of medically compromised patients by means of dental implants. In a case series of 24 patients (16 females, 8 males) with special treatment requirements (systemic diseases, congenital defects), including a female patient with SSc and Sjogren’s syndrome, 103 implants and the corresponding prosthetic rehabilitation were followed up over a time scale of between 2 and 12 years. The cumulative survival rate for these inserted implants was 97.3% during the healing phase and 93.4% for 63 implants that could be examined after a period of five years [42]. In a retrospective study, the implants (*n* = 89) were subjected to a follow-up analysis in 22 patients suffering from autoimmunologically induced rheumatic diseases (rheumatoid arthritis, collageneses), including a patient with SSc. The cumulative survival rate for 21 implants that could still be followed up in the fifth year of observation amounted to 96.1% [45]. When compared with peri-implant parameters of bone loss, pocket depth, plaque index, gingiva index, bleeding index and CPI that were continually collected, the patients with rheumatoid arthritis and those with collagenoses ultimately suffered from increased bone resorption and an increased bleeding index, although this was not significant.

Seven single case reports were published between 1990 and 2016 detailing the implant surgery and prosthetic restoration of patients (totalling 6 females and 1 male) with SSc. The described examinations indicated that a minimum of 2 and a maximum of 12 implants were inserted. For six cases, the survival rate for prosthetically burdened implants was 100% during the observation period of 24–60 months (Table 14) [42, 45–49].

**Table 14:** Summary of publications concerning the implantological treatment of patients with systemic sclerosis

Publication	Casuistic	Case series	Age	Gender	Implan-t(s)	Survival rate (%)	Observation period (Months)
Baptist (2016) [49]	x		61	f	6	100	30
Zigdon et al. (2011) [48]	x		45	f	12	100	36
Weinländer et al. (2010) [45]		(1 × SSc)	-	f	6	100	46
Öczakir et al. (2005) [42]		(1 × SSc)	64	f	8	100	60
Haas et al. (2002) [67]	x		49	f	7	N.A	N.A
Patel et al. (1998) [68]	x		54	f	4	N.A	N.A
Raviv et al. (1996) [47]	x		65	f	3	100	28
Langer et al. (1992) [69]	x		54	f	2	N.A	N.A
Jensen u. Sindet-Pedersen (1990) [46]	x		39	m	9	88.9	24

Regarding SSc, little published evidence is available regarding the long-term survival of inserted implants, as the relevant studies are single case reports. A bias attributable to positive selection (highly motivated and therapy-adherent patients, treatment under idealised conditions with a high time requirement) should also be considered in view of the roughly comparable results of a meta-analysis of results from casuistics with an incidence rate of 0.031 implant losses per year in patients with SSc.

A systematic review on the implant treatment of patients with oral mucosa changes included an analysis of the oral rehabilitation of patients suffering from SSc by means of dental implants. A weighted mean implant survival rate of 97.4% (± 4.8 SD) was determined on the basis of five single case reports that met the inclusion criteria for 38 inserted implants over a mean follow-up examination duration of 38.3 months (± 13.4 SD). The opinion of the authors was that no clinical recommendations could be given for the treatment of patients with SSc in relation to implants. Among other recommendations, the authors advised interdisciplinary decision-making measures based on personalised medicine to establish an indication for implant therapy [43]. In another systematic review regarding implants in patients with autoimmune or oral mucosa diseases, the success of implant treatment was examined in patients with SSc. Six SSc patients (mean age 54.9 ± 10.7) with 44 implants could be included in the statistical analysis on the basis of the inclusion criteria. A mean follow-up observation period of 37.5 ± 13.4 months resulted in a weighted mean implant survival rate of 97.7% (± 15.1 SD) [44].

In our patient cohort, a post-10-year implant survival rate of 87.6% (95%-KI: 75.5–94.0) was calculated in the statistical analysis for 20 SSc patients with 65 implants (Fig. 4). To the best of our knowledge, this is the numerically largest cohort with the longest published observation period (mean 5.9 years) in connection with implants in patients with SSc to date. The development of the PLI, SBI and PPD values under restricted opening of the mouth and disease-related manual impairments are of special interest.

Our examinations revealed that none of the SSc patients were subjected to a PLI of 3 in terms of implant prosthetic restoration during an observation period of 10 years. Only PLIs 0, 1 and 2 were discernible during the follow-up examinations. The determination of the SBI primarily resulted in indices 0 and 1. Index 2 was only determined during four of the ten observation periods. An SBI of 3 was not registered at any time during the examinations. The PLI indices only significantly deviated from the initial examination during three of the ten observation periods, with a difference in the SBI determinations being registered in six of the ten observation periods (Tables 4 and 5). The PPD measurements resulted in a mean value between 2.4 and 2.8 mm with no significant differences during the entire observation period (Table 6). As signs of inflammation and an increase in probing depths were evident only during a few patient visits, radiographs were not routinely taken. This procedure is in accordance with the German S3 guideline "The treatment of peri-implant infections on dental implants" [50].

**Fig. 5:**
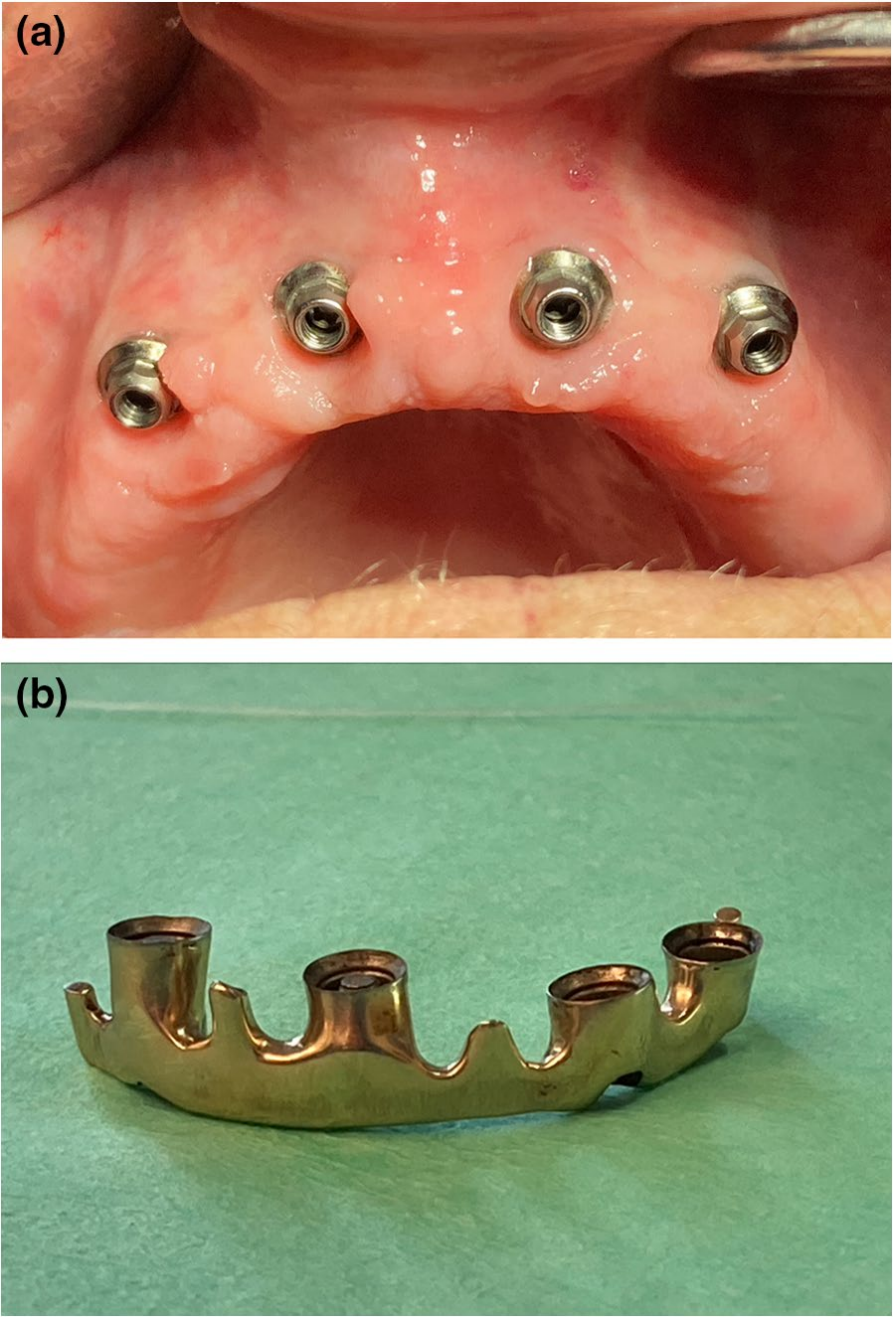
**a** Peri-implant gingival situation in an 80-year-old patient with SSc eleven years after incorporation of an implant-bar-fixed denture of the maxilla. **b** Follow-up (same patient, **a**): 11 years in function. Implant bar directly after removal, no accumulation of plaque

When one considers all three parameters in context, significant positive correlations can be shown between the PLI and the SBI values on the one hand (Table 7) and between the PLI values and the PPD values (Table 8) on the other. The correlation is, however, pronounced to a greater extent between PLI and SBI than between PLI and PPD (Tables 7, 8 and 9). The results of the statistical analysis enable the conclusion to be drawn that the patients with SSc that were included in this case series were highly aware of the importance of adequate oral hygiene as a factor for the survival of the implant. Despite restricted manual skills, the SBI, the PLI, and the PPD all remained within ranges that did not risk the survival of the implants during each of the observation periods (Fig. 5a, b).

With regard to the peri-implant parameters of bone loss, pocket depth, plaque index, gingiva index, bleeding index and CPI, which were continually determined during the follow-up examinations, a comparison of female patients with rheumatoid arthritis with those having collagenoses showed that the latter displayed increased bone resorption and a higher bleeding index, although this was not significant [45]. The PPD was stated as being 3.2 ± 2.1 mm in the 29 patients with collagenoses. As a comparison of these results is possible with our findings, we obtained, during a period of 10 years (Table 6), mean PPD values of between 2.4 mm (SD 0.6 mm) and 2.8 mm (SD 0.7 mm), which is quite comparable to data from implant studies with healthy subjects [51].

SSc can present itself orally with microstomia, a CPI score of > 2, an increased DMFT index, inadequate oral hygiene, a reduced salivary flow rate and a reduced pH value, xerostomia, oral telangiectasia, periodontal microcirculation disorders, a widening of the periodontal gap, mandibular resorptions, bone resorptions and trigeminal neuralgia, among other symptoms [52–55]. In a periodontological study, 20 SSc patients and 20 controls were subjected to PD (probing depth), PLI, GI (gingival index) and BOP (bleeding on probing) examinations. TNF-α (tumour necrosis factor-alpha) was also measured in the gingival crevicular fluid (GCF). Higher indices for periodontal inflammatory procedures and TNF-α values were determined in the SSc patients than in the control group [56]. In a descriptive case–control study, 50 SSc patients were compared with 43 healthy patients. A significantly higher frequency of periodontitis was observed in the SSc patients than in the control group (90.7 × 48.83%; *p* < 0.001). The typical pattern of periodontal disease in the SSc group included a low probing pocket depth (2 ± 0.65 mm × 2 ± 0.24; *p* < 0.001) and a lower gingival bleeding index value (7.05 ± 7.25 × 21.57 ± 15.66; p < 0.001) [31].

The PLI, SBI and PPD values that we have determined with regard to the implants during the follow-up examinations do not show increased indices for periodontal inflammatory processes in SSc patients relative to those for natural teeth obtained in other studies.

The Canadian Systemic Sclerosis Oral Health Study III included 163 SSc patients and 231 controls. This is the largest study conducted on a group of SSc patients in which diverse oral health parameters were determined based on the care of standardised dental examination. This study was unable to establish a connection between periodontopathy in SSc patients and the degree of severity of SSc. However, it did establish that a reduction in the interincisal gap was related to the severity of the disease and that a correlation existed with the values determined from the modified Rodan skin thickness score. The number of teeth that were missing was associated with reduced saliva production, gastroesophageal reflux disease and restricted mobility affecting the hands [57]. In contrast to the Canadian Systemic Sclerosis Oral Health Study III, the statistical analysis of a comparative study with 58 SSc patients and 52 controls resulted in the determination of a possible connection between SSc and periodontitis [32].

Digital ulcerations also manifest themselves on the fingertips, digital furrows, and extension side of the joints in connection with calcinoses in SSc patients. Approximately 30% of patients with SSc develop such ulcerations each year, causing pain, functional impairment, disfiguration and a considerably reduced quality of life [58]. In another study, 80 patients suffering from SSc (67 females, 13 males; mean age: 53.4 ± 11.7) were subjected to clinical and radiological examinations to determine possible correlations in terms of sex, interstitial lung disease (ILD), modified Rodnan skin score (mRSS), fingertip to palm (FTP) distance and IED. The ID was significantly lower for females than for males, and it was also lower in the diffuse type of SSc than in the limited type (p < 0.001 und p < 0.001). Furthermore, it was significantly lower for the patients with ILD than for those without ILD (p = 0.006). Significant negative correlations were also noted between the ID and the mRSS and between the ID and the FTP distance (p < 0.001 and p < 0.001) [59]. SSc-related contraction of the perioral tissue with the resulting microstomia is related to a reduced quality of life because food intake is made considerably more difficult and oral hygiene cannot be adequately carried out. In the Canadian SSc oral health study, patients with SSc had a larger number of destroyed teeth than was the case for patients who were not suffering from SSc (163 SSc patients and 231 controls, SSc 0.88, controls 0.59, P = 0.0465). The SSc patients also produced less saliva (SSc 147.52 mg/min, controls 163.19 mg/min, P = 0.0259). Oral health-related quality of life (HRQoL) was significantly reduced compared with that of the healthy controls (mean OHIP score: SSc 41.58, controls 26.67, P < 0.0001). This is the reason that prosthetic rehabilitation in the form of a conventional or an implant-supported dental prosthesis is of such importance [30]. The integration and removal of conventional prosthetic dental prostheses can be rendered more difficult or even impossible in patients with SSc. Dental/oral surgical intervention is also impaired, as the posterior areas of the oral cavity are not always accessible. Prosthetic treatment of some patients with microstomia cannot be carried out without surgical widening of the corner of the mouth (commissurotomy); this is especially the case if the circumference of the mouth is smaller than 160 mm [60–62]. An important point that should be taken into account in connection with such surgery is that wound healing impairments can result in a manifestation of scars leading to a recurrence of microstomia, thereby counteracting the actual surgical objective. A therapeutic alternative to commissurotomy is the use of foldable or dismountable dental prostheses that can be easily removed as they are made of supple silicone and provided with attachments or hinges allowing them to be connected to each other to give rigidity. After they have been incorporated into the oral cavity, the components can later be removed in the event of the oral opening becoming restricted [63–65]. A 65-year-old male patient with microstomia as a result of burns was treated with a foldable complete-mandible dental prosthesis that comprised two separate components produced using a casting technique [62]. Such prosthetic options as described in the literature for microstomia work as long as the patient has no manual impairments. As far as our SSc patients were concerned, the disease-related changes to the hands and fingers were the reasons that we dispensed with the possibility of using a foldable prosthesis when a corresponding indication existed. Customisation of the treatment of patients with hyposalivation/xerostomia is possible. Instead of an implant-supported total prosthesis, consideration should be given to the therapeutic alternative of a shortened row of teeth that is screwed to implants.

Many oral manifestations, including xerostomia, microstomia, decreased vascularity, bone resorption, and tooth mobility, may affect the choice and survival of dental implants in SSc patients. Microstomia, bone resorption, and potential adjacent tissue reaction to implants may be detrimental to the provision of implant technology in edentulous SSc patients. As SSc is an autoimmune multisystem rheumatic disease affecting connective tissue, and an inflammatory, vascular and sclerotic disease of the skin, the oral mucosa as well as of several organs (lung, heart, gastrointestinal tract), especially involvement of perioral and oral tissues will limit the prosthetic therapy with any type of removable restoration [44]. However, the principles of osseous and soft tissue regeneration are basically not different between a healthy patient and a patient with SSc. However, it should be noted that the accompanying circumstances, such as reduced mouth opening, may increase the difficulty of surgical treatment. In addition, fibrosis of the perioral dermis leads to reduced elasticity of the corners of the mouth, which often makes it difficult to visualise the surgical area well. However, damage to neighbouring structures (for example, tearing of the corners of the mouth) must be avoided, for example, by using petroleum jelly. It is also often not possible to insert implant drills axially, so there is a need to work with short implants or angled abutments. Close perioperative collaboration between the oral surgeon or the oral and maxillofacial surgeon (OMFS) is crucial in this field of dental implantology. As far as the fabrication of the prosthetic denture is concerned, it must be ensured that the patient is also able to insert and remove it properly. Regardless of whether the prosthesis is designed to be fixed or removable, the patient must be able to independently ensure the longevity of the implant-supported restoration through home oral care. Regular check-ups at semiannual intervals should be aimed at detecting any pathological changes. In many cases, the fibrosis process in the perioral area progresses rapidly so that removable dentures often have to be reduced in dimension to remain functional. In general, it can be said that the treatment of SSc patients belongs in the hands of an experienced oral surgeon or OMFS, and close cooperation between dental professionals, rheumatologists or dermatologists is necessary for decision-making, treatment planning and maintenance.

Although the patients included in our study have considerable impairments regarding the opening of their mouths and their manual skills, the determined PLI, SBI, and PPD values together with the survival rate of the implants indicate that the patients have a high degree of motivation and subject the implant emergence points and the implant prosthetic restoration to effective oral hygiene.

## Conclusions

In view of the presented extraoral and intraoral symptom complexes, we consider that SSc patients require a carefully coordinated dental medicine/oral surgery therapy concept to retain or restore chewing function. One reason for this is that the patients need to eat numerous smaller meals with large quantities of fluid each day. Food should also be chewed thoroughly because of possible disease-related motility disturbances that affect the oesophagus. Hyposalivation or xerostomia, microstomia and/or restricted manual skills that are typical for SSc cause considerable difficulties with daily oral hygiene and in the incorporation and excorporation of removable dental prostheses. SSc patients can gain discernible benefits from the insertion of implants to replace single teeth or to support removable dental prostheses. These can necessitate the use of highly customised prosthetic solutions. Implant-supported rehabilitation of the masticatory organ is a successful therapeutic option when taking the existing casuistics into account. The indication for implant therapy should be established within the scope of an interdisciplinary consultation with consideration being given to the individual situation of the SSc patient. A prerequisite for long-term implant survival is a high degree of patient motivation that forms the basis for the acceptance of undertaking regular intraoral follow-up examinations.

## Data Availability

The datasets used and/or analysed during the current study are available from the corresponding author on reasonable request.

## Abbreviations

ACR: American College of Rheumatology
EULAR: European League against Rheumatism
CI: Confidence interval
dSSc: Diffuse systemic scleroderma
IED: Incisal edge distance
lSSc: Limited systemic scleroderma
OMFS: Oral and Maxillofacial Surgeon
PLI: Plaque Index
PPD: Pocket probing depth
SBI: Sulcus Bleeding Index
SSc: Systemic scleroderma
TMD: Temporomandibular disorders
